# Sequential Anaerobic–Aerobic Treatment Enhances Sulfamethoxazole Removal: From Batch Cultures to Observations in a Large-Scale Wastewater Treatment Plant

**DOI:** 10.1021/acs.est.4c00368

**Published:** 2024-07-08

**Authors:** Caglar Akay, Nadin Ulrich, Ulisses Rocha, Chang Ding, Lorenz Adrian

**Affiliations:** † Department Molecular Environmental Biotechnology, Helmholtz Centre for Environmental Research − UFZ, Permoserstraße 15, 04318 Leipzig, Germany; ‡ Department Exposure Science, Helmholtz Centre for Environmental Research − UFZ, Permoserstraße 15, Leipzig 04318, Germany; § Department Applied Microbial Ecology, Helmholtz Centre for Environmental Research − UFZ, Permoserstraße 15, Leipzig 04318, Germany; ∥ Chair of Geobiotechnology, Technische Universität Berlin, Ackerstraße 76, Berlin 13355, Germany

**Keywords:** antibiotics, biotransformation, transformation products, sulfate-reducing condition, nitrate-reducing condition, wastewater treatment

## Abstract

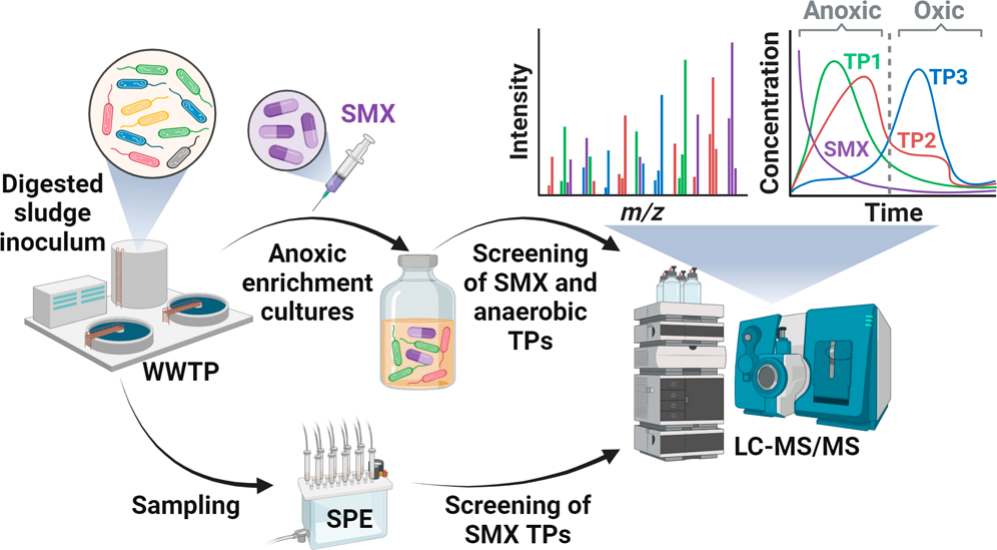

Sulfamethoxazole (SMX) passes through conventional wastewater treatment plants (WWTPs) mainly unaltered. Under anoxic conditions sulfate-reducing bacteria can transform SMX but the fate of the transformation products (TPs) and their prevalence in WWTPs remain unknown. Here, we report the anaerobic formation and aerobic degradation of SMX TPs. SMX biotransformation was observed in nitrate- and sulfate-reducing enrichment cultures. We identified 10 SMX TPs predominantly showing alterations in the heterocyclic and *N*^4^-arylamine moieties. Abiotic oxic incubation of sulfate-reducing culture filtrates led to further degradation of the major anaerobic SMX TPs. Upon reinoculation under oxic conditions, all anaerobically formed TPs, including the secondary TPs, were degraded. In samples collected at different stages of a full-scale municipal WWTP, anaerobically formed SMX TPs were detected at high concentrations in the primary clarifier and digested sludge units, where anoxic conditions were prevalent. Contrarily, their concentrations were lower in oxic zones like the biological treatment and final effluent. Our results suggest that anaerobically formed TPs were eliminated in the aerobic treatment stages, consistent with our observations in batch biotransformation experiments. More generally, our findings highlight the significance of varying redox states determining the fate of SMX and its TPs in engineered environments.

## Abstract 2

This study reports the anaerobic transformation of sulfamethoxazole, the detection of transformation products and the aerobic degradation and prevalence of these transformation products in real wastewater treatment plants.

## 1 Introduction

Sulfamethoxazole (SMX) is a widely used broad-spectrum antibiotic, known for contributing to the development and spread of antibiotic resistance.^1,2^ Frequent occurrence of SMX in various environmental compartments, often in combination with numerous other compounds and many unknown transformation products (TPs), raises emerging concerns. These combinations can create a potent chemical cocktail with harmful effects on aquatic organisms in the receiving environments.^3,4^

Wastewater treatment plants (WWTPs) function as crucial receptors and emitters of diverse pharmaceuticals in large quantities, significantly influencing the fate of SMX and its TPs before being discharged into surface waters. Reported SMX concentrations have been as high as 1340 μg/L in wastewater from pharmaceutical industries,^5^ 8.84 μg/L in livestock wastewater effluent,^6^ 27.8 μg/L in hospital wastewater,^7^ 7.91 μg/L in municipal WWTP influents, and 9.46 μg/L in WWTP effluents,^8^ indicating incomplete SMX removal in conventional WWTPs with removal efficiencies varying from 0 to 90%, depending on the processes and operational conditions employed.^9^ In Swiss WWTPs, 60–80% of SMX together with its human metabolite, *N*^4^-acetyl-SMX, were removed by a conventional activated sludge process and a combined anaerobic-anoxic-oxic membrane bioreactor.^10^ Primary clarifiers did not contribute significantly to SMX removal, and sorption to sludge played a minor role since SMX has a low affinity for sorption to solid surfaces, thus leading to high mobility in aqueous environments.^11,12^ Consequently, SMX has been detected at maximum concentrations of 16.7 μg/L in surface waters, 1.11 μg/L in groundwater, 0.116 μg/L in drinking water, and 671.5 μg/kg in soils,^4,13^ with geographical variations depending on the extent of consumption and availability of waste management systems. A recent study revealed significantly higher SMX concentrations in surface waters in Africa and South America compared to those in Europe and North America.^4^

Microbial transformation has been reported as the main removal mechanism of SMX in natural and engineered systems. Microorganisms capable of transforming SMX aerobically under laboratory conditions include species of *Rhodococcus*,^14,15^*Microbacterium*,^16^*Pseudomonas*,^17,18^*Acinetobacter*,^19^*Achromobacter*,^20,21^*Sphingobacterium*,^22^ and *Shewanella.*(18) In most of these studies, biotransformation was reported to occur via two major routes: in route 1, the S–N bond of the sulfonamide group was initially cleaved, yielding sulfanilamide and 3-amino-5-methylisoxazole (3A5MI). While sulfanilamide was partially or completely degraded, 3A5MI accumulated as a dead-end product. In route 2, the aniline group was modified by substitution of a hydrogen residue with an acetyl, hydroxyl, or formyl group. Recently, the formation and occurrence of pterin-conjugated TPs of SMX as well as their retained antibiotic activity were reported in activated sludge systems in Swiss WWTPs.^23^

Other studies emphasize the significance of different redox conditions for SMX removal.^11,24−26^ The impact of varying electron-accepting processes, e.g., aerobic, nitrate-reducing, iron(III)-reducing, and sulfate-reducing conditions, was investigated on the fate of SMX in soil microcosms.^25^ Iron(III)-reducing conditions was the most favorable for SMX transformation, whereas no SMX was transformed under nitrate-reducing conditions. The detected TPs were isomerized, reduced, and hydroxylated forms of SMX with alterations observed only in the isoxazole moiety. In batch cultures with sediment under nitrate-reducing conditions, *N*^*4*^-nitro-SMX and desamino-SMX were detected as TPs with retransformation of *N*^*4*^-nitro-SMX back to SMX.^27^ Similarly, *N*^*4*^-nitro-SMX formation under nitrate-reducing conditions with aquifer material.^28^ Another study reported SMX biotransformation under anoxic conditions with reductive cleavage taking place at the isoxazole moiety.^11^ In sulfate-reducing batch cultures and a bioreactor, several SMX TPs with modified isoxazole moiety were detected.^29^ Recently, we reported microbial transformation of SMX in sulfate-reducing and methanogenic mixed cultures as well as with a sulfate-reducing pure strain of *Desulfovibrio vulgaris* Hildenborough.^26^ In that study, isomerized and reduced TPs were identified.

While information on aerobic SMX biotransformation and TPs formed under anoxic conditions in laboratory cultures is available, little is known about the fate of anaerobic TPs and whether these anaerobic biotransformation processes occur in WWTPs. Therefore, the goal of the current study was to investigate anoxic transformation reactions of SMX to identify characteristic TPs and to evaluate (i) if these TPs can be fully degraded under subsequent aerobic conditions and (ii) if such TPs occur in real WWTPs. We report on the microbial transformation of SMX in various anoxic conditions. We analyzed the microbial community compositions under two electron-accepting processes and determined the effect of different electron donors, the formation of TPs, and the stability of TPs under oxic conditions. Finally, we detected the occurrence of the same anaerobic TPs in different stages of a full-scale municipal WWTP in Leipzig, Germany.

## 2 Material and Methods

### 2.1 Cultivation of Enrichment Cultures

The defined mineral medium contained the following minerals: 200 mg/L KH_2_PO_4_, 270 mg/L NH_4_Cl, 1000 mg/L NaCl, 410 mg/L MgCl_2_·6H_2_O, 520 mg/L KCl, and 150 mg/L CaCl_2_·2H_2_O. In addition, we added 1 mL/L of a trace element solution (1.5 mg/L FeCl_2_·4H_2_O, 0.07 mg/L ZnCl_2_, 0.1 mg/L MnCl_2_·4H_2_O, 0.006 mg/L H_3_BO_3_, 0.19 mg/L CoCl_2_·6H_2_O, 0.002 mg/L CuCl_2_·2H_2_O, 0.024 mg/L NiCl_2_·6H_2_O, 0.036 mg/L Na_2_MoO_4_·2H_2_O) and 1 mL/L of a Se/W Solution (500 mg/L NaOH, 6 mg/L Na_2_SeO_3_·5H_2_O and 8 mg/L Na_2_WO_4_·2H_2_O). Finally, 0.1% (w/v) Na-resazurin was amended as a redox indicator. The medium was sparged with N_2_ for 60 min and transferred into serum bottles inside an anaerobic chamber (Coy Laboratory Inc., USA) containing a gas phase of 97% N_2_ and 3% H_2_. The bottles were sealed with butyl rubber septa and aluminum crimp caps within the anaerobic chamber and then autoclaved. The following components were added from sterile stock solutions into the autoclaved serum bottles: 10 mM NaHCO_3_, 1 mL/L vitamin solution (20 mg/L biotin, 20 mg/L folic acid, 252 mg/L pyridoxine-HCl, 50 mg/L riboflavin, 50 mg/L thiamine-HCl, 50 mg/L nicotinic acid, 50 mg/L Ca-d-pantothenate, 50 mg/L *p*-aminobenzoic acid, 50 mg/L cyanocobalamin, and 50 mg/L α-lipoic acid) and 2 mM l-cysteine (except for nitrate-reducing conditions). K_2_SO_4_ and NaNO_3_ were added as electron acceptors at 1 mM concentrations for sulfate-reducing and nitrate-reducing conditions, respectively. As for electron donors, treatment 1 (T1) was amended with 3 mM Na-l-lactate, treatment 2 (T2) with 1 mM Na-acetate and 4 mM H_2_, and treatment 3 (T3) contained no additional electron donor or carbon source. SMX was spiked at a nominal concentration of 700 nM. Anaerobic digested sludge from a municipal WWTP (Klärwerk Rosental) in Leipzig, Germany, was used as seed inoculum to establish enrichment cultures (1%, v/v). The cultures were passaged into fresh media always using a 10% (v/v) inoculum and amended with 700 nM of SMX at each passage. Abiotic controls without cultures (NCC, “no cell control”), control cultures without SMX (NSC, “no SMX control”) and autoclaved cultures with SMX (sorption) were prepared in parallel. The bottles were incubated at 30 °C in the dark without shaking and all experiments were performed in triplicates.

### 2.2 Analytical Methods

Targeted screening for SMX: samples were analyzed with an Agilent 1260 Infinity II Series liquid chromatography (LC) system coupled to an AB Sciex QTRAP 6500+ tandem mass spectrometer (MS/MS) equipped with a Turbo V ion source. The system was operated with electrospray ionization in positive polarity. Chromatographic separation was achieved with an Agilent Zorbax Eclipse Plus Rapid Resolution HT-C18 (100 mm × 3.0 mm, 1.8 μm) column with a Phenomenex Security guard cartridge (C18; ODS, Octadecyl). The injection volume, flow rate, and column temperature were 20 μL, 0.4 mL/min, and 30 °C, respectively. The mobile phase consisted of 0.2% formic acid in LC-MS grade water (solvent A) and methanol (solvent B). The gradient elution was as follows: 0–2 (10% of B), 2–3 (10–60% of B), 3–8 (60–90% of B), 8–11 (90% of B), and 11.1–16 min (10% of B). Quantitative analysis was performed using multiple reaction monitoring (MRM) mode, with MRM transitions optimized by direct infusion of the standard solutions (Table S1). All data was acquired and processed using Analyst 1.7.2. software.

Suspect screening for unknown TPs: screening for unknown TPs of SMX was performed using a precursor ion (PI) survey scan that generated typical sulfonamide fragment ions of *m*/*z* 92, 108, and 156. The PI scan was combined with information-dependent acquisition (IDA) criteria set at an intensity threshold of 5,000 cps, followed by an enhanced product ion (EPI) scan as the dependent scan. When a precursor ion was detected above the specified intensity threshold, an EPI scan was triggered to acquire a full-scan MS/MS spectrum across the *m*/*z* range of 55 to 550 to obtain fragment data. The PI spectra of samples were acquired by scanning over the *m*/*z* range of 90–550 at a scan rate of 200 Da/s using a declustering potential at 55 V, entrance potential at 10 V, collision energy at 20 V, and collision cell exit potential at 10 V. The EPI spectra were obtained at a scan rate of 10 000 Da/s using dynamic fill time at a declustering potential of 55 V, an entrance potential of 10 V, a collision energy of 35 V, and a collision energy spread of 15. The gradient elution was the same as the MRM method. Semiquantitative determination of the detected TPs at each sampling interval was done by using extracted ion chromatograms (XIC) of each detected TP at an *m*/*z* range window of ±0.5. Normalization of peak areas of each TP was done by dividing the peak area of the selected TP at a specific time interval by the maximum peak area observed for the selected TP during the experiment. Data acquisition and processing was done using Analyst 1.7.2 Software. Tentative identification of TPs and possible biotransformation reactions were assessed using SCIEX-OS-Q v2.2.0 and Lightsight v2.3.1 software, respectively.

### 2.3 Wastewater Treatment Plant Samples

One liter samples of raw influent, primary clarifier effluent, biological treatment effluent, final effluent, and digested sludge of a municipal WWTP (Klärwerk Rosental) located in Leipzig, Germany, were collected in December 2022 and stored at +4 °C for 2 weeks until sample preparation. The samples were centrifuged and filtered through 0.22 μm polyethersulfon membrane filter units and enriched by solid phase extraction (SPE) using Oasis HLB (6 cc, 200 mg) cartridges from Waters (Milford, USA). The cartridges were preconditioned with 5 mL of methanol followed by 10 mL of 0.1 M NaCl solution. The pretreated 1 L samples were spiked with 1 μg/L SMX-d4, loaded onto the cartridges, and then washed with 0.1 M NaCl. The analytes were eluted two times with 2 mL of a mixture of methanol/water/formic acid (50:50:0.1, v/v/v) for LC-MS/MS analysis. The samples were then analyzed in predictive MRM (*p*MRM) mode combined with IDA criteria and EPI scan, which includes predicted MRM transitions of the previously detected TPs. SMX and *N*^4^-acetyl-SMX were quantified on the basis of neat standards using internal standard-based normalization (eq 3). Concentrations of other TPs were estimated based on a semiquantification approach (eq 4).^30−33^ The details of *p*MRM list are provided in Table S2.

### 2.4 Data and Statistical Analysis of SMX Biotransformation

One-way ANOVA with the Bonferroni multiple comparison test was conducted to analyze the statistical differences of removal efficiencies among different electron donor treatments for each redox condition using SigmaPlot 14.5 software. The *p*-values less than 0.05 were considered statistically significant.

First-order reaction kinetics was used to assess SMX biotransformation (eq 1):
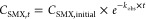
1where *C*_SMX,initial_ is the initial SMX concentration (nM), *C*_SMX,*t*_ is SMX concentration at the specific time interval (*d*) and *k*_obs_ is the observed biotransformation rate constant (d^–1^).

The removal efficiency (%) of SMX was calculated using the eq 2:
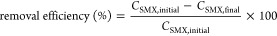
2where *C*_SMX,final_ is the SMX concentration at the last time interval (*d*).

Internal standard-based normalization of SMX concentration (eq 3)
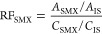
3

Semiquantitative analysis of TPs without authentic standards (eq 4)
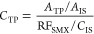
4where RF_SMX_ is the response factor of SMX, *A* is the peak area, and *C* is the concentration. IS and TP stand for internal standard and transformation product (TP), respectively.

## 3 Results and Discussion

### 3.1 Effect of Electron Donors on SMX Biotransformation under Nitrate-Reducing Conditions

**Figure 1:**
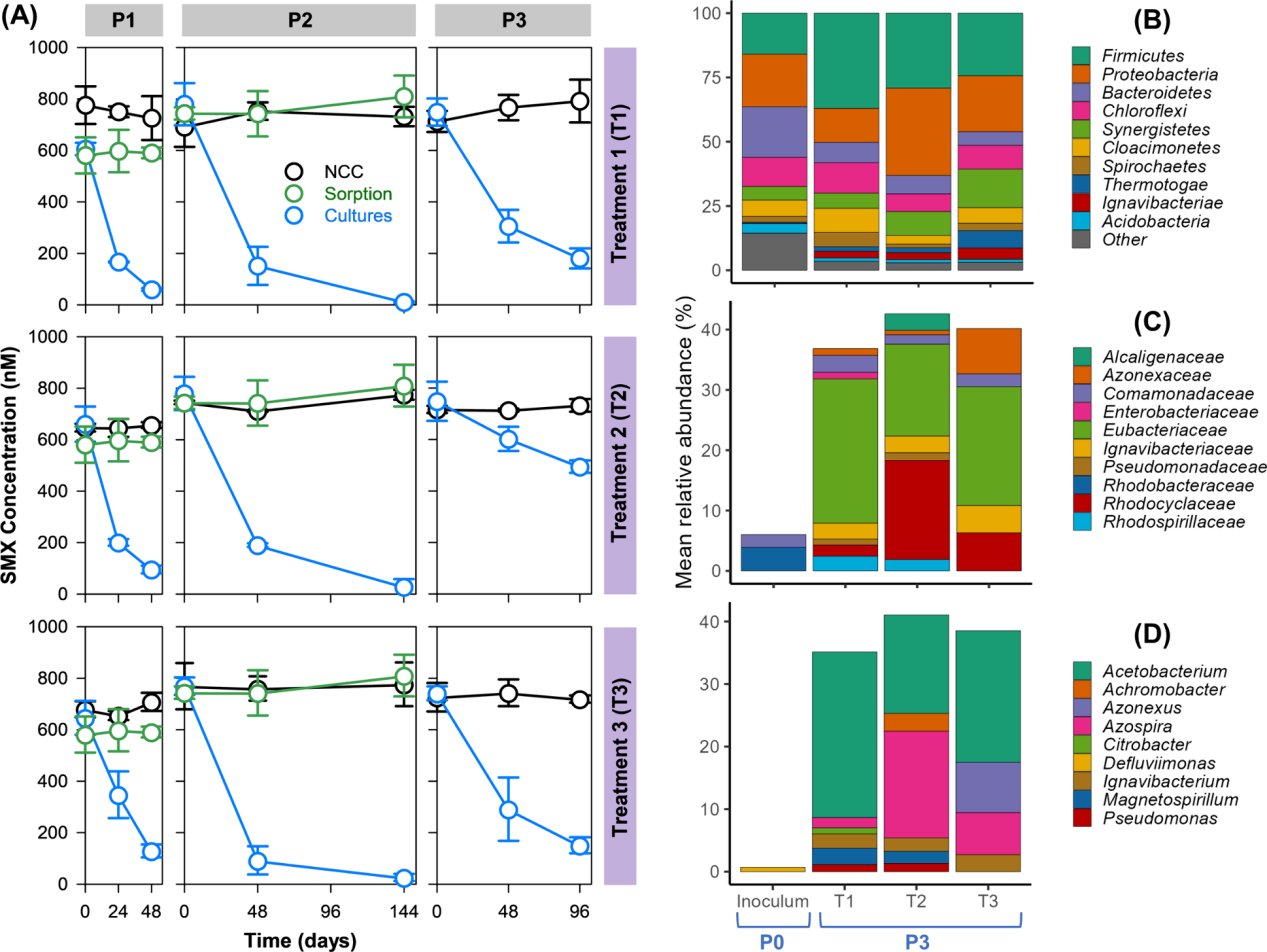
(A) SMX biotransformation under nitrate-reducing conditions in the presence of different electron donors. Treatment 1 (T1) contains 3 mM lactate, T2 contains 1 mM acetate plus 4 mM H2, and T3 was without an additional electron donor. P1, P2, and P3 denote enrichment passages 1, 2, and 3, respectively. Data show means and standard deviations, *n* = 3. Panels (B–D) show the mean relative abundance of taxonomic groups in cultures from P0 and P3. Taxonomic levels are shown as phyla (B), families (C), and genera (D). Only the 10 most abundant taxa are displayed at the phylum level, with the rest labeled as others. Taxa with relative abundances exceeding 0.5%, which have been described as capable of nitrate reduction, are shown at the family and genus levels.

SMX biotransformation was investigated under nitrate-reducing conditions with different electron donor treatments including 3 mM lactate as treatment 1 (T1), 1 mM acetate in combination with 4 mM H_2_ as T2, and no additional electron donor/carbon source as treatment (T3). By transferring 10% of inoculum into fresh medium for three successive passages, microbial transformation was demonstrated as the main process for SMX removal (Figure 1A). SMX transformation was not observed in any of the abiotic controls (NCC) or autoclaved sorption controls. This suggests that SMX was chemically stable in the defined mineral medium, and sorption did not account for the loss of SMX during the incubations. SMX removal efficiencies by enriched cultures in the third passage (P3) were 76 ± 5% for T1, 34 ± 5% for T2, and 80 ± 4% for T3 after 96 days. The mean values of the observed first-order rate constants (*k*_obs_) were estimated as 0.013 (T1), 0.004 (T2), and 0.018 d^–1^ (T3) with the coefficient of determination (*r*^2^) values of 0.989, 0.999, and 0.996, respectively. The biotransformation of SMX was significantly lower (*p* < 0.001) in T2 compared to the other two treatments. Ion chromatography results of P3 cultures revealed that NO_3_^–^ was completely removed after 96 days, confirming nitrate-reduction activity in all cultures. These findings are in contrast to the previously published study, demonstrating negligible SMX biotransformation in a soil microbial community in the presence of 25 μM SMX, 5 mM NO_3_^–^, and 3.13 mM acetate.^25^ Similarly, SMX transformation was not observed under nitrate-reducing enrichments established with wetland sediment or anaerobic digested sludge amended with 1 mM NO_3_^–^ and 700 nM SMX.^26^ On the other hand, in water-sediment batch experiments under denitrifying conditions supplemented with higher NO_3_^–^ concentrations (67 mM), approximately 4 μM SMX was transformed within 10 days of incubation. The *k*_obs_ values estimated in our study were lower than those of the estimated *k*_obs_ value (0.2 d^–1^).^27^

To identify microbial communities, we performed Illumina sequencing of 16S rRNA gene amplicons and analyzed the taxonomic assignments in the digested sludge inoculum (P0) and at the end of passage 3 (P3) samples with T1, T2, and T3 treatments. At phylum level, *Firmicutes* and *Proteobacteria* were the most predominant populations in all treatments (Figure 1B). Among 24 bacterial families known to be capable of reducing nitrate, the 10 most dominant families with relative abundances above 0.5% are shown in Figure 1C. The relative abundance of the *Comamonadaceae* family did not change significantly from passage 0 to passage 3. The *Rhodobacteraceae* family was detected only in the inoculum, and was not enriched during cultivations. In contrast, the relative abundances of *Eubacteriaceae*, *Rhodocyclaceae*, *Azonexaceae*, and *Ignavibacteriaceae* increased in all P3 treatments. All SMX-transforming enrichments consistently contained the genera of *Acetobacterium*, *Azospira*, and *Ignavibacterium* at high population shares (Figure 1D), suggesting that bacteria from these genera are involved in SMX transformation under nitrate-reducing conditions.

### 3.2 Effect of Electron Donors on SMX Biotransformation under Sulfate-Reducing Conditions

**Figure 2:**
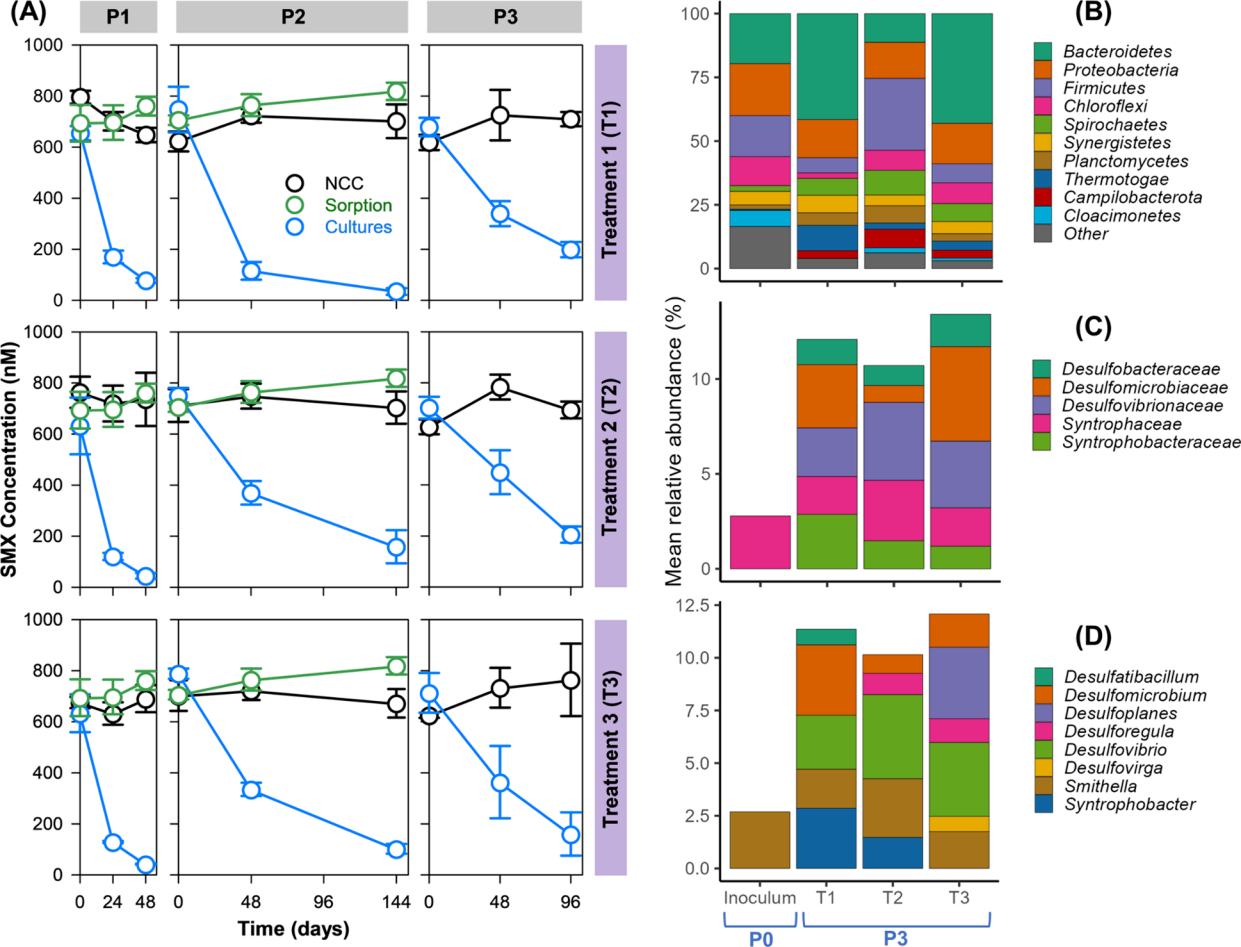
(A) SMX biotransformation under sulfate-reducing conditions in the presence of different electron donors. Treatment 1 (T1) contains 3 mM lactate, T2 contains 1 mM acetate plus 4 mM H2, T3 was without an additional electron donor. P1, P2, and P3 stand for enrichment passages 1, 2, and 3, respectively. Data show means and standard deviations, *n* = 3. Panels (B–D) show the mean relative abundance of taxonomic groups in cultures from P0 and P3. Taxonomic levels are shown as phyla (B), families (C), and genera (D). Only the 10 most abundant taxa are displayed at the phylum level, with the rest labeled as others. The predominant sulfate-reducing bacterial families and genera with relative abundances above 0.5% are shown at the family and genus levels.

We then investigated SMX biotransformation with the same electron donor treatments in sulfate-reducing conditions as well. Similar to the setup under nitrate-reducing conditions, SMX was abiotically stable and the removal was not linked to sorption in all electron donor treatments (Figure 2A). The cultures maintained their SMX-transforming activity in three successive passages. The activity of cultures was confirmed by measuring sulfate via ion chromatography. SMX removal efficiencies were found to be 71 ± 4%, 71 ± 3%, and 77 ± 12% for T1, T2, and T3, respectively. The first-order rate constants were estimated as 0.013 d^–1^ (T1), 0.011 d^–1^ (T2), and 0.015 d^–1^ (T3) with the coefficient of determination (*r*^2^) values of at least 0.979 for all three treatments. Unlike under nitrate-reducing conditions, the rates and removal efficiencies of SMX biotransformation were not significantly affected by different electron donor treatments. The estimated *k*_obs_ values for sulfate-reducing enrichments in our study were similar to those,^26^ which were between 0.009 and 0.015 d^–1^, however, significantly lower than the *k*_obs_ value of 0.11 d^–1^ reported in sulfate-reducing batch cultures.^29^

In all sulfate-reducing treatments, the majority of microbial phyla consisted of *Bacteroidetes*, *Proteobacteria*, and *Firmicutes* (Figure 2B). From P0 to P3, the relative abundance of OTUs associated with sulfate-reducing bacterial families significantly increased, with the exception of the *Syntrophaceae* family, which remained relatively the same (Figure 2C). The predominant sulfate-reducing bacterial families, all of which belong to the *Proteobacteria* phylum were *Desulfovibrionaceae*, *Desulfomicrobiaceae*, *Desulfobacteraceae*, and *Syntrophobacteraceae*. The genera of *Desulfomicrobium* and *Desulfovibrio* were constantly present in all SMX-transforming enrichments (Figure 2D). Previously, SMX-transforming activity of *Desulfovibrio vulgaris* Hildenborough strain belonging to the *Desulfovibrio* genus was reported.^26^ Thus, bacteria from the aforementioned four sulfate-reducing bacterial families enriched during cultivation might also be potential SMX transformers.

### 3.3 Identification of Transformation Products (TPs)

To investigate the effect of electron donor treatments on the formation of SMX TPs under nitrate- and sulfate-reducing conditions, we monitored them in P3 cultures of all electron donor and acceptor treatments after six months of cultivation. TPs were not detected in the cell-free (NCC) and SMX-free (NSC) controls, implying that the formation was associated with microbial transformation. Information on the observed masses and fragments of TPs, retention times (R_t_), tentative elemental compositions, proposed structures and the corresponding treatments are given in Table 1.

**Table 1:**
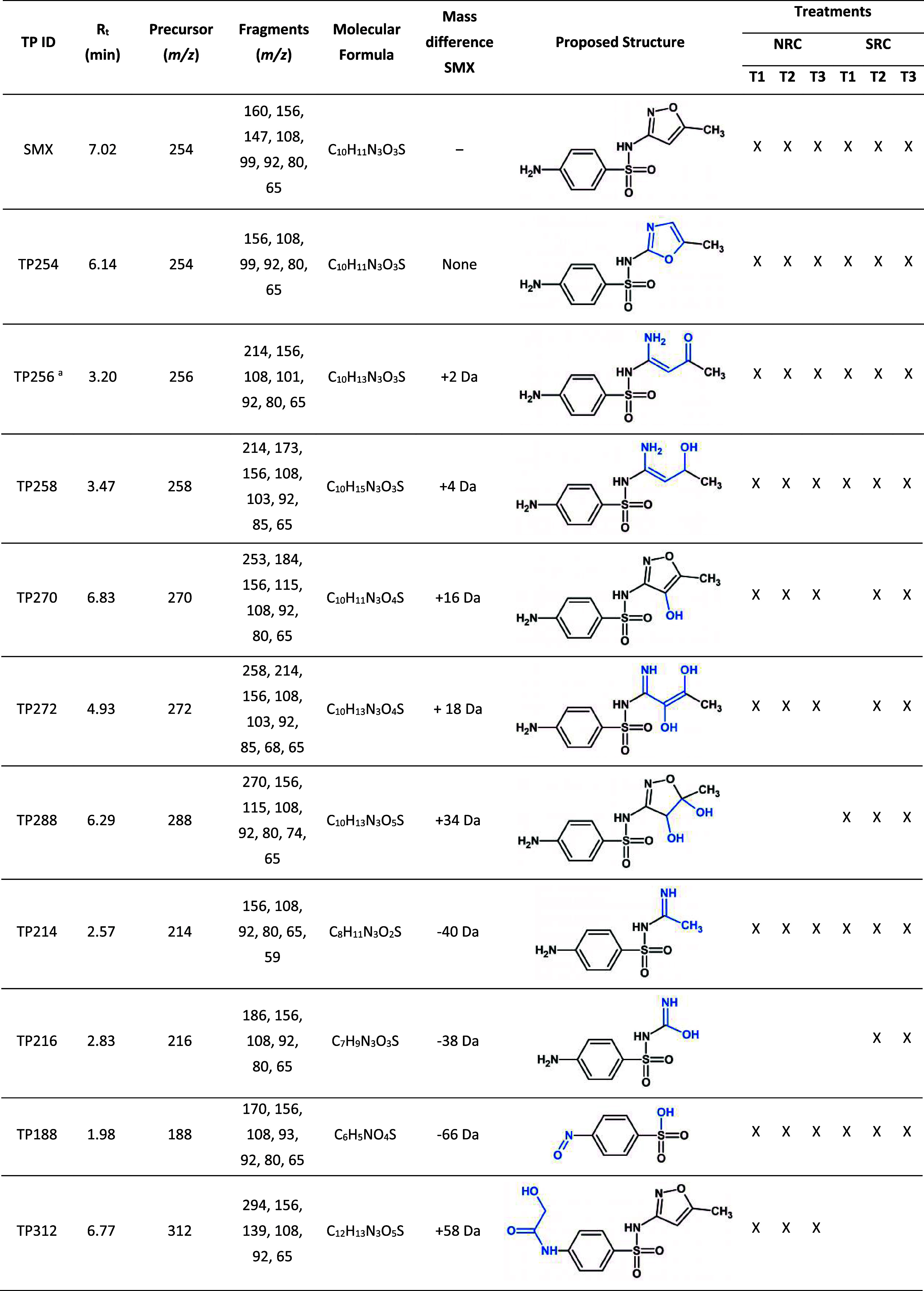
Detected Anaerobic Transformation Products (TPs) of SMX in the P3 Enrichment Cultures^b^

a TP256: proposed structure shown here is the reduced and cleaved isoxazole ring of SMX. TP256 might also be formed via the same reaction taking place in oxazole moiety of TP254.

b NRC and SRC stand for nitrate-reducing and sulfate-reducing conditions, respectively.

The protonated SMX (*m*/*z* = 254) as the parent ion yields typical sulfonamide fragment ions at *m*/*z* 92, 108 and 156, as well as *m*/*z* 99 from the heterocyclic isoxazole moiety known as 3A5MI (Figure S1). By monitoring this characteristic fragmentation pattern, we identified common fragment ions in mass spectra of TPs, thus elucidating tentative structures.

TP254 (*m*/*z* = 254) showed the same fragmentation pattern as we observed with SMX, but eluted earlier during chromatographic separation, indicating an isomerization reaction of the isoxazole moiety to oxazole (Figure S2A). The product with a mass shift of +2, TP256 (*m*/*z* = 256), exhibited the same fragments as SMX except for *m*/*z* 99 ion. The presence of the fragment ion at *m*/*z* 101 indicated a reduction of either the isoxazole or oxazole moiety (Figure S2B). These two TPs were previously reported in microbially mediated iron-reducing soil microcosms,^25^ anaerobic sulfate-reducing sludge system,^29^ and in mixed and isolated sulfate-reducing bacteria.^26^ The dihydrogenated product, TP258 (*m*/*z* = 258), could have been formed via the reduction of TP256. Existence of the fragment ion at *m*/*z* 103 was in accordance with the double reduction of SMX (Figure S2C). A similar tentative structure was proposed for TP258 as an SMX biotransformation product under anaerobic conditions, using skimmed milk and bicarbonate as the carbon source and electron acceptor.^11^ The same TP was also observed both in biologically active and sterilized anaerobic sludge.^34^ The fragmentation of TP270 (*m*/*z* = 270) resulted in fragment ions with *m*/*z* values of 156, 115, 108, 92, 80, and 65 (Figure S2D). The absence of *m*/*z* 99 and the presence of *m*/*z* 115 revealed that a hydroxylation reaction with a mass shift of +16 took place in the isoxazole moiety. TP270 was detected in all treatments except T1 conducted under sulfate-reducing conditions. The detection of this product is consistent with the study, investigated SMX biotransformation in an anaerobic packed bed bioreactor.^35^ Another hydroxylation product, TP288 (*m*/*z* = 288), showed identical fragments of TP270 with the additional fragment ion at *m*/*z* 270 (Figure S2F). Thus, this product was confirmed to be dihydroxylated SMX and expected to be formed via subsequent hydroxylation of monohydroxylated TP270. The dihydroxylated SMX has been frequently detected in various advanced oxidation processes,^36−39^ also identified in soil enriched with poultry manure,^40^ and in lab-scale biofilters amended with acetate and manganese oxide.^41^ However, its identification as a microbial TP of SMX under sulfate-reducing conditions was reported only once in the literature.^29^ To our best knowledge, this is the second report of TP288 observed in sulfate-reducing enrichments. On the contrary, TP288 was not detected in any of the nitrate-reducing treatments. In addition, we also detected TP272 at *m*/*z* 272 (Figure S2E). This product could be formed via the hydroxylation of reduced and cleaved isoxazole moiety of TP258. Similar to TP270, TP272 was observed in all treatments except T1 under sulfate-reducing conditions.

We attributed the formation of TP214 to the loss of an acetyl group (−C_2_H_3_O) in the cleaved isoxazole moiety of TP256 (Figure S2G). This claim is supported by the fragment ions at *m*/*z* 59 for TP214 and at *m*/*z* 214 for TP256. The MS^2^ spectrum of TP216 (*m*/*z* = 216) suggests subsequent demethylation (mass shift of −14) and oxidation (mass shift of +16) reactions of TP214 (Figure S2H). Another possibility for the formation of TP216 is the loss of −C_3_H_4_ (mass shift of −40) in the cleaved and reduced isoxazole moiety of TP256. TP216 was reported in an anaerobic sulfate-reducing sludge system,^29^ as well as in plant tissues from soil fertilized with manure.^42^ TP216 was only observed to be formed in T2 and T3 under sulfate-reducing conditions. Furthermore, we detected another product, TP188 (*m*/*z* = 188), with fragment ions at *m*/*z* 170, 156, 108, 93, 92, 80, and 65 (Figure S2I). The precursor ion indicates an odd number of nitrogen atoms for this product. We suggest that TP188 could be formed via the cleavage of the S–N bond on the sulfonyl-amine group (−SO_2_–NH_2_) upon H_2_O attack, resulting in the formation of sulfanilic acid (−SO_3_H). The *N*^4^-arylamine moiety of sulfanilic acid would then undergo stepwise hydroxylation and autoxidation reactions at the *N*-terminus to form 4-hydroxyaminobenzene-1-sulfonic acid and 4-nitrosobenzene-1-sulfonic acid, respectively. The observation of fragment ions at *m*/*z* 170 and 93 supports this hypothesis. Lastly, we detected TP312 (*m*/*z* = 312) only in nitrate-reducing enrichments (Figure S2J). The mass shift of +58 indicates an acetylation reaction (+42) took place in the *N*^4^-arylamine moiety to form *N*^4^-acetyl-SMX, followed by hydroxylation (+16) in the same moiety of *N*^4^-acetyl-SMX. This hypothesis is supported by the fragment ion at *m*/*z* 294. However, we did not detect *N*^4^-acetyl-SMX as the intermediate product of this transformation. Contrary to the previous reports,^27,28^*N*^*4*^-nitro-SMX and desamino-SMX under nitrate-reducing conditions were not observed in our study.

It is evident from the proposed structures of the detected TPs, the *N*^4^-arylamine moiety remained intact and did not undergo transformation (except for TP188 and *N*^4^-acetyl-SMX). The reactions took place at the isoxazole moiety both under nitrate-reducing and sulfate-reducing conditions. This phenomenon is not surprising since isoxazoles contain a weak N–O bond, which is readily cleaved under reductive conditions.^43^ It should be noted that the heterocyclic isoxazole byproducts 3A5MI, monohydroxylated-3A5MI, and dihydroxylated-3A5MI were not detected in this study.

### 3.4 Fate of the Transformation Products under Oxic Conditions

**Figure 3:**
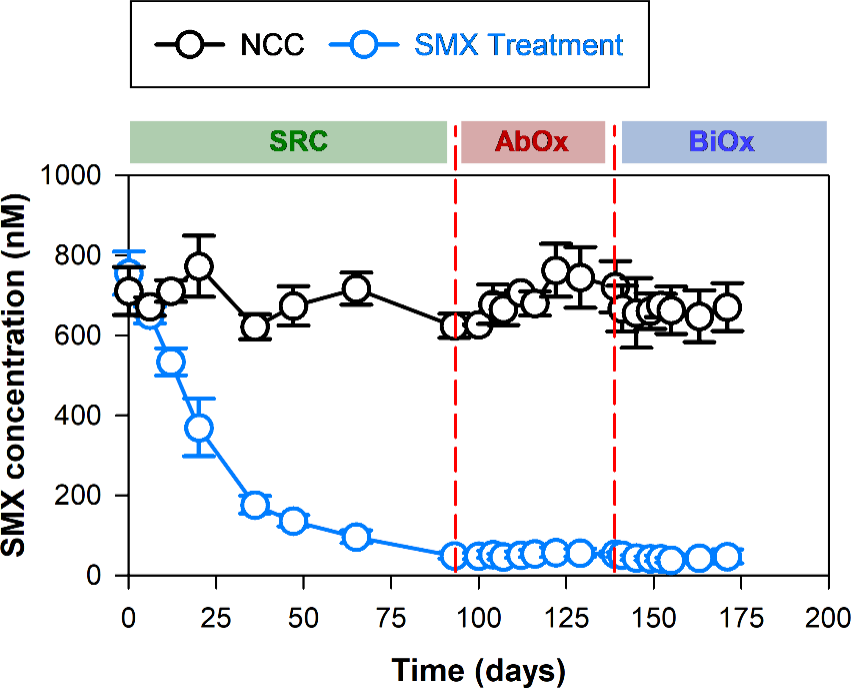
SMX biotransformation with the cultures from passage 4 (P4) under sulfate-reducing conditions (SRC) followed by abiotic-oxic (AbOx) and biotic-oxic (BiOx) treatments. Values represent means ± standard deviations, *n* = 3.

**Figure 4:**
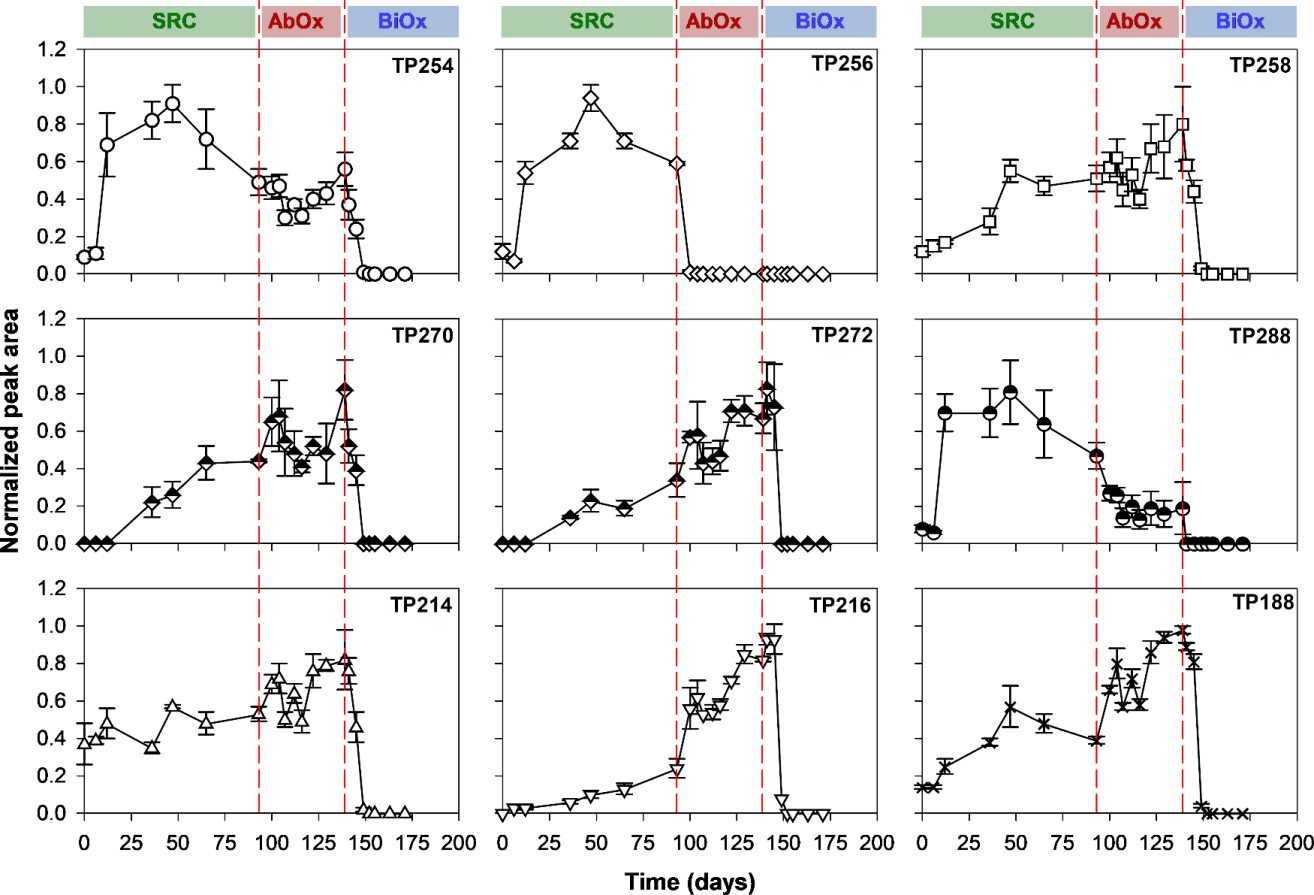
Time course evolution of transformation products (TPs) with successive sulfate-reducing (SRC), abiotic-oxic (AbOx), and biotic-oxic (BiOx) treatment stages. Values represent means ± standard deviations, *n* = 3. For better visualization, normalized peak areas are shown. Normalized peak areas are defined as the peak area of a TP at a specific time interval divided by the maximum peak area observed for the selected TP during the experiment. Absolute peak area counts are listed in the Supporting Information Table S4.

As demonstrated above, SMX was transformed into a variety of TPs that were stable under nitrate- and sulfate-reducing conditions. We then assessed stability of these TPs under oxic conditions. For this, anaerobic formation of SMX TPs was replicated by transferring 10% (v/v) of the nitrate-reducing or sulfate-reducing P3 cultures from treatment 3 into fresh medium amended with 1 mM nitrate or sulfate, and spiked with 700 nM of SMX. Freshly inoculated nitrate-reducing and sulfate-reducing P4 cultures were incubated at 30 °C in the dark without shaking for 59 and 93 days, respectively. SMX removal efficiencies were found as 24 ± 3% for nitrate reducing (Figure S3) and 93 ± 1% for sulfate-reducing cultures (SRC) (Figure 3). Due to comparatively slow transformation of SMX in nitrate-reducing enrichments in P3, the two sequential oxic treatments were conducted only with sulfate-reducing enrichments. At day 93, sulfate-reducing P4 cultures were sterile-filtered through 0.22 μm cellulose acetate filters into sterile Erlenmeyer flasks and agitated on an orbital shaker at 85 rpm at 20 °C in the dark for 46 days (here referred to as “abiotic-oxic treatment”, AbOx). Afterward, the culture filtrates were aseptically amended with 0.1% (v/v) vitamin solution, reinoculated with 1% (v/v) digested sludge, and incubated at 20 °C in the dark for 32 days (referred to as “biotic-oxic treatment”, BiOx). The evolution of the previously detected TPs was monitored throughout all three incubation stages (Figure 4).

Abundance of the TPs based on their detected peak area counts over the course of three sequential incubation stages were in the following order (data based on PI scan with *m*/*z* 156 fragment): TP256 > TP254 > TP258 > TP188 > TP288 > TP216 > TP214 > TP272 > TP270. During the cultivation under sulfate-reducing conditions, SMX concentrations decreased continuously over time while the peak area counts of the two most abundant TPs, TP254 and TP256, increased correspondingly until day 47 (Figure 4A,B). After day 47, the formation of TP254 and TP256 stopped, and the peak area counts started to decrease, suggesting that they were further transformed while they were not further produced from SMX due to the low SMX concentrations after day 47. During AbOx, the isomerized TP254 remained stable. On the other hand, the reduced TP256 diminished quickly within 7 days and was removed entirely from the system. The secondary reduced product TP258 formed more slowly during cultivation than TP256 and TP254 and remained stable during AbOx stage (Figure 4C). The formation of dihydroxylated TP288 (Figure 4F) followed a similar formation pattern to TP256, whereas the monohydroxylated TP270 (Figure 4D) slowly increased over time under sulfate-reducing conditions. Similar to TP270, the formation of the other monohydroxylated TP272 was also slow in sulfate-reducing conditions (Figure 4E). During AbOx, TP288 was observed to be decreased to a level in which it remained almost constant prior to BiOx. On the contrary, peak area counts of TP270 and TP272 showed increasing patterns during AbOx. TP188 was demonstrated to be formed during sulfate-reducing conditions. During AbOx, peak area counts of TP188 (Figure 4I) significantly increased, suggesting formation via the oxidation of TP256, which was unstable when exposed to oxygen. Similarly, TP216 emerged significantly during AbOx and increased until the reinoculation under oxic conditions (Figure 4H). Detected area counts of TP188 and TP216 were significantly higher (*p* < 0.001) in AbOx treatment than in sulfate-reducing conditions, indicating their emergence through oxidative transformation of anaerobic TP(s). The detection of both under sulfate-reducing conditions, however, could be due to sample preparation conducted under oxic conditions. After reinoculation and incubation under oxic conditions (BiOx), all the detected TPs were removed completely within 6 days and were not observed again during the incubation. Additionally, none of the TPs were transformed back to SMX. Our observations show that SMX biotransformation was achieved under anoxic conditions, resulting in the formation of several anaerobic TPs with altered isoxazole moieties. Some of these TPs were unstable during the period of abiotic exposure to oxygen (AbOx) and were transformed to secondary TPs. Subsequent reinoculation and incubation under oxic conditions (BiOx) demonstrated that anaerobic TPs of SMX were completely removed from the system without transforming back to SMX. Therefore, we conclude that the persistence of SMX can be greatly reduced by anaerobic pretreatment and could potentially reduce the toxicity of SMX.

### 3.5 Monitoring of Wastewater Treatment Plant Samples for SMX and Suspected TPs

**Figure 5:**
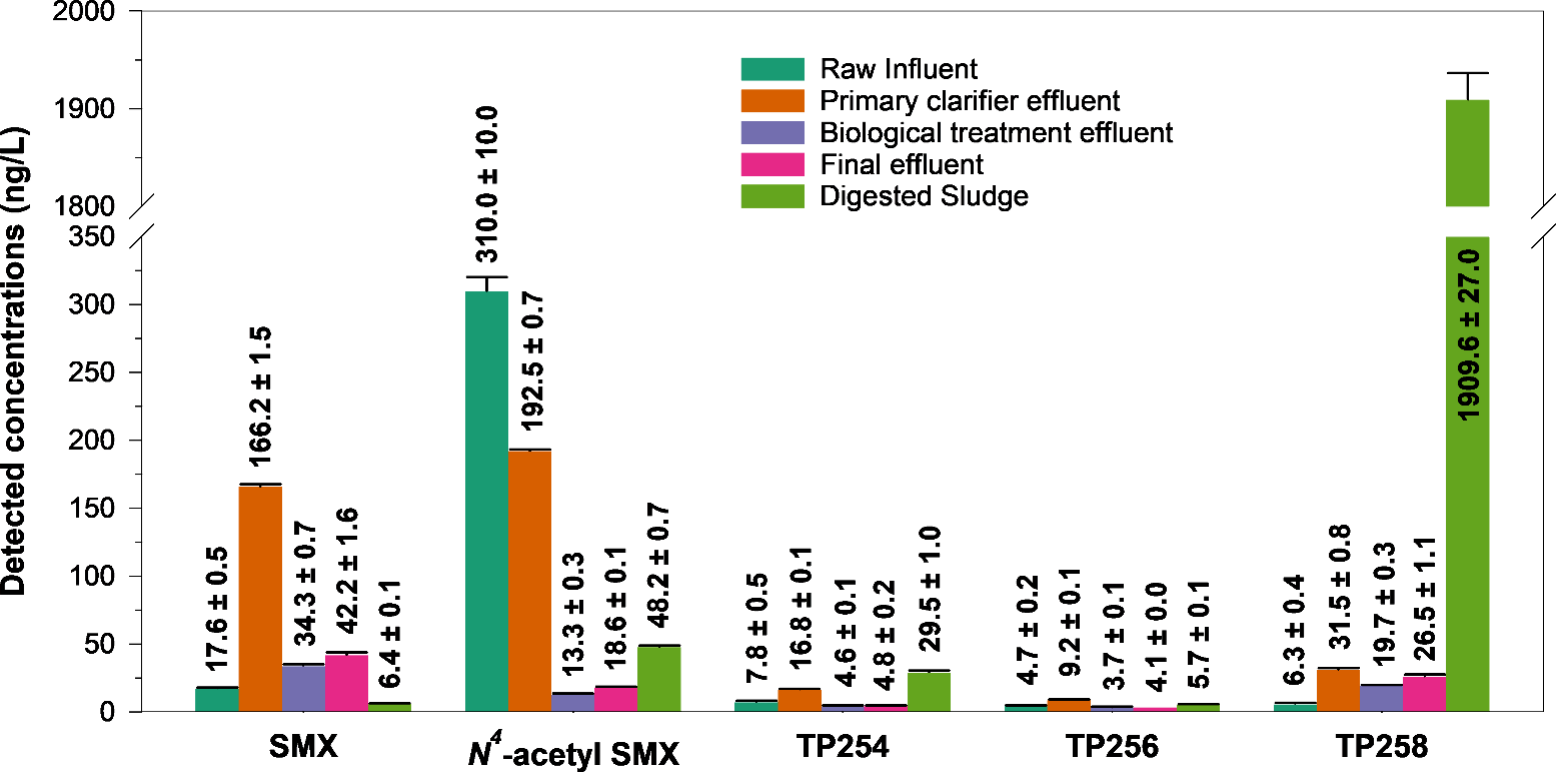
Detected concentrations of SMX, N4-acetyl-SMX, and TPs in various treatment processes of Rosental WWTP. Values represent means ± standard deviations of the technical replicates, *n* = 3. Concentrations of TPs were estimated using the semiquantification approach, based on the assumed structural similarity between TPs and SMX as illustrated in eq 4.

We finally investigated the relevance of the observed anaerobic biotransformation of SMX in a conventional municipal WWTP. For that, collected samples from different stages of a WWTP in Leipzig, Germany (Figure S4), were analyzed by LC-MS/MS to detect and quantify SMX, *N*^4^-acetyl-SMX and previously observed anaerobic TPs of SMX (Figure 5). In the raw influent of the WWTP, the mean concentration of SMX was lower than in the primary clarifier effluent; however, much higher concentrations of the human metabolite, *N*^4^-acetyl-SMX, were detected in the raw influent. In the primary clarifier unit, SMX concentrations were increased by nearly 9.4-fold compared to the raw influent, while mean *N*^4^-acetyl-SMX removal efficiency was 38%. The anaerobic TPs also exhibited increased concentrations between the raw influent and the primary clarifier effluent, with mean production values of 54% for TP254, 49% for TP256, and 80% for TP258. In the biological treatment unit, receiving primary clarifier effluent, up to 80% of SMX and 93% of *N*^4^-acetyl-SMX were removed. Here, the maximum removal efficiencies of TPs were 73, 61, and 40% for TP254, TP256, and TP258, respectively. The concentrations of SMX, *N*^4^-acetyl-SMX and anaerobic TPs in the final effluent were slightly higher than in the biological treatment effluent. The lowest SMX concentration was detected in the digested sludge, while TP258 and TP254 concentrations were detected at the highest. The concentration of *N*^4^-acetyl-SMX in the digested sludge was 2.6-fold greater than in the final effluent.

The presence of *N*^4^-acetyl-SMX in high concentrations in the raw influent suggests that the WWTP Rosental receives SMX primarily in its acetylated form. This finding is expected considering 40–70% of SMX is excreted from the human body as *N*^4^-acetyl-SMX.^44^ The WWTP Rosental is one of the oldest WWTPs in Germany and receives the wastewater of about 550,000 inhabitants of the Leipzig urban area. During pretreatment in the primary clarifier, *N*^4^-acetyl-SMX was transformed back into its parent form, as previously reported in a Swiss WWTP.^10^ Previously, *N*^4^-acetyl-SMX and SMX were detected in the primary clarifier effluent of WWTP Rosental at median concentrations of 2300 and 1000 ng/L, respectively.^45^ Detection of three anaerobic TPs in the primary clarifier effluent indicates the prevalence of anoxic conditions. Concentrations of these TPs decreased during the biological treatment, where oxic conditions prevailed. In the digested sludge, which received sludge from the primary and secondary clarifiers, all anaerobic TPs were detected at high concentrations. Notably, TP258 and TP254 eluted later than TP256 during chromatographic separation, indicating that they may be more hydrophobic, leading to their accumulation in digested sludge. In batch experiments, TP254 and TP258 remained stable under anoxic and abiotic-oxic conditions but degraded by microbial processes under oxic conditions, as was also demonstrated by their occurrence in the anoxic/oxic stages of the WWTP. Several studies documented the abundance and activity of sulfate-reducing bacteria in various stages of municipal WWTPs, including the pipelines of sewage systems, primary clarifiers, digested sludge, and aerated activated sludge units.^46−50^ Therefore, sulfate-reducing bacteria might have contributed to the anaerobic biotransformation of SMX, as evidenced by the detection of anaerobic TPs in anoxic stages of the WWTP. Exceptionally high concentrations of TP258 in digested sludge samples could be attributed to ion enhancement caused by the complex matrix effect during measurement. Additionally, the semiquantitative approach may be susceptible to increased errors due to unknown ionization efficiency of TPs. Our observations indicate that anaerobic SMX transformation, even though restricted to anoxic stages in real WWTPs, serves as an important bioprocess for its detoxification and subsequent degradation in the receiving compartments. It may be necessary to take such bioprocess into consideration when designing or upgrading WWTPs, in order to achieve concurrent removal of contaminants of concern.

In our study, we observed microbial transformation of SMX under nitrate- and sulfate-reducing conditions, regardless of different electron donor amendments. The lower redox potential under sulfate-reducing conditions favored SMX biotransformation more than nitrate-reducing conditions. Anaerobic SMX biotransformation was initiated at the isoxazole moiety, whereas the aromatic *N*^4^-arylamine moiety was not altered with the exception of two transformation products (TPs). Sequential aerobic treatments further led to the degradation of anaerobically formed TPs of SMX, thus leading to the reduction of persistence and toxicity. While anaerobic SMX biotransformation have been previously reported in laboratory-scale experiments, this study represents the first documented occurrence of these processes in a full-scale municipal wastewater treatment plant (WWTP). We demonstrated that anaerobic biotransformation plays a significant role in the removal and detoxification of SMX in the receiving environments. Overall, our findings emphasize the importance of the interplay between anoxic and oxic conditions on the fate of SMX and its TPs in engineered environments.

## Supporting Information Available

Supplementary materials and methods: chemicals and reagents; nitrate and sulfate measurements; microbial community analysis; QSAR analysis of TPs; supplementary results: ecotoxicity assessment of the detected TPs; Tables S1–S4: details of MRM transitions; list of *p*MRM transitions; QSAR analysis of SMX and its TPs; absolute peak area counts of the detected TPs; Figures S1–S4: mass spectrum of SMX; mass spectra of the detected TPs; SMX biotransformation under nitrate-reducing with cultures from enrichment passage 4; simplified process diagram and operational details of WWTP Rosental (PDF)
