# Supplementary material for: Sequential Anaerobic–Aerobic Treatment Enhances Sulfamethoxazole Removal: From Batch Cultures to Observations in a Large-Scale Wastewater Treatment Plant
Source: Environ Sci Technol. 2024 Jul 8;58(28):12609–20. doi: 10.1021/acs.est.4c00368 (PMC11256761; doi:10.1021/acs.est.4c00368)
Supplement: Supplementary file 1 — es4c00368_si_001.pdf [file es4c00368_si_001.pdf]

Supporting Information

Sequential anaerobic-aerobic treatment enhances sulfamethoxazole removal: From batch cultures to observations in a large-scale wastewater treatment plant

Caglar Akay[1], Nadin Ulrich[2], Ulisses Rocha[3], Chang Ding[1], Lorenz Adrian[1, 4*]

[1]Helmholtz Centre for Environmental Research – UFZ, Department Molecular Environmental Biotechnology, Permoserstraße 15, 04318 Leipzig, Germany

[2]Helmholtz Centre for Environmental Research – UFZ, Department Exposure Science, Permoserstraße 15, 04318 Leipzig, Germany

[3]Helmholtz Centre for Environmental Research – UFZ, Department Applied Microbial Ecology, Permoserstraße 15, 04318 Leipzig, Germany

[4]Chair of Geobiotechnology, Technische Universität Berlin, Ackerstraße 76, 13355 Berlin, Germany

*Lorenz Adrian, Helmholtz Centre for Environmental Research – UFZ, Molecular Environmental Biotechnology, Permoserstraße 15, 04318 Leipzig, Germany, Tel.: +49 341 6025 1435, E-Mail: lorenz.adrian@ufz.de

This supplement contains 15 pages including Supplementary methods (2 pages), Supplementary results (1 page), 4 tables, 4 figures and Supplementary references

Supplementary materials and methods

Chemicals and reagents

The analytical grade standards of sulfamethoxazole (SMX), its human metabolite *N*^{4}-acetylsulfamethoxazole (*N*^{4}-acetyl-SMX) and deuterium labeled internal standard sulfamethoxazole-d4 (SMX-d4) were purchased from Sigma-Aldrich (Taufkirchen, Germany) with purities of at least 98%. LC-MS grade methanol and water were obtained from ChemSolute (Renningen, Germany), whereas formic acid was purchased from Serva Electrophoresis GmbH (Heidelberg, Germany). All other chemicals used for mineral culture medium were obtained from Merck (Darmstadt, Germany) with purities of at least 98%.

Primary stock solutions of analytes were prepared in methanol. Working standard solutions were obtained by dilutions of the stock solutions in methanol. The working standard solutions were further diluted in a mixture of methanol/water/formic acid (20:80:0.1, v/v/v) to prepare a series of calibration standards. Stock and working standard solutions were stored in amber vials at −20°C and prepared every six months. The calibration standards were prepared freshly at each measurement interval.

Measurement of nitrate and sulfate with ion chromatography

Nitrate and sulfate concentrations were measured using a Dionex ICS-5000 ion chromatography system (Thermo Scientific, Germany) equipped with a Dionex IonPac AS18 column (2 mm × 150 mm, Thermo Scientific, Germany). Potassium hydroxide and milli-Q water were used as the mobile phase with a flow rate of 0.25 mL/min. The gradient elution was as follows: 0-2 min at 10 mM, 2-28 min ramp up from 10 to 40 mM, 28–32 min ramp up from 40 to 55 mM, and 32–36 min at 10 mM. Detection of nitrate and sulfate was achieved with a UV detector at a wavelength of 210 nm, and a conductivity detector, respectively.

Microbial community analysis

Genomic DNA was extracted from 2 mL samples of enrichment cultures at passage 0 (digested sludge inoculum) and passage 3 (P3) of all electron acceptor and donor treatments by using NucleoSpin® tissue kit (Macherey-Nagel, Germany) according to the manufacturer's protocol. DNA concentrations were quantified using Qubit® 3.0 fluorometer for double-stranded-DNA high sensitivity assay kit (Thermo Fischer Scientific, Germany). Extracted DNA was stored at −80°C and sent on dry ice to Eurofins Genomics Europe Sequencing GmbH (Konstanz, Germany). The 16S rRNA PCR amplification, barcoding and Illumina sequencing were performed by Eurofins Genomics. DNA samples were subjected to barcoded primer PCR amplification of the region V3-V4 using the bacterial primer pair 341F (5′ CCTACGGGNGGCWGCAG 3′) – 785R (5′ GACTACHVGGGTATCTAATCC 3′). Library construction and MiSeq sequencing (300 bp read length) was carried out at Eurofins Genomics. The raw sequences were deposited in the NCBI SRA database under the BioProject PRJNA989073 (https://www.ncbi.nlm.nih.gov/sra/PRJNA989073). Paired-end MiSeq reads were merged using the fastq_merge-pairs option from the USEARCH software package version 11.0.667[1] with a minimum

identity of 60% and a maximum mismatch number of 60 bp for overlapping regions. Primers were trimmed from both edges of the assembled read pairs using fastx_truncate from USEARCH. Quality filtering was applied using fastq_filter from USEARCH with a maximum expected error number of 1.0. After filtering, there were between 63,411 and 95,832 clean reads for each sample in the dataset. Unique sequences were picked using fastx_uniques from USEARCH. Operational taxonomic unit (OTU) picking from unique sequences was done at a clustering identity of 100% using the unoise3 option from USEARCH and chimeras were removed during this step. OTU tables were generated using the otutab option from USEARCH[2]. Taxonomies were assigned to OTUs by the RDP Naive Bayesian rRNA Classifier Version 2.11 and RDP 16S rRNA training set 16 (bootstrap threshold 80%) on the online server (http://rdp.cme.msu.edu/)[3]. Data curation and visualization of taxonomic composition were done in R using *tidyverse*, *dplyr* and *ggplot2* packages.

Ecotoxicological assessment of TPs with QSAR

Quantitative structure-activity relationship (QSAR) analysis was done using VEGA QSAR (https://www.vegahub.eu) to assess the ecotoxicological hazard of SMX TPs. The tentative chemical structures of TPs were drawn using ACD/ChemSketch 2020.1.2 Software (Toronto, Canada). As input parameters in VEGA, we used simplified molecular input-line entry system (SMILES) notations of SMX and TPs. By applying the KNN read-across model, we determined the bioconcentration factor (BCF). LC_{50} (48 h) for *Daphnia magna*, and LC_{50} (96 h) for *Fathead minnow* were determined according to the EPA model. EC_{50} (72 h) for *Pseudokirchneriella subcapitata* as algae acute toxicity were determined by ProtoQSAR-Combase prediction model.

Supplementary results

Ecotoxicity assessment of the observed TPs with QSAR analysis

In the last step of our study we used QSAR modelling to predict the potential toxicity of the observed TPs of SMX (Table S3). The predicted BCF of SMX and the observed TPs were in the range of 0.1 to 1.5 log unit. These values are well below the described log BCF threshold of 3.7, where molecules considered to be highly bioaccumulative. Therefore, SMX and the observed TPs are not likely to accumulate in selected aquatic organisms. The predicted 48 h LC_{50} values for *Daphnia magna* for SMX and TPs were 13.2–709.7 mg/L with the lowest values predicted for TP254 and TP214, and the highest values for the hydroxylated TPs. The 96 h LC_{50} values for *Fathead minnow* were in the range of 8.7–579.5 mg/L. Here, the highest value was predicted for TP288, and the lowest for TP188. The 72 h EC_{50} values for *Pseudokirchneriella subcapitata* (algae acute toxicity) were predicted at 0.1037–374.1 mg/L. The lowest value was predicted for TP312, whereas the highest for TP214, TP216, and TP188, which were three orders of magnitude higher than those of other TPs. However, the reliability of the prediction outcomes was defined as low. Therefore, the estimated values should only be used as an indicator for the potential hazard of TPs since experimental data is not available, and the training set of structurally similar compounds used in QSAR analysis are limited. SMX had a 72 h EC_{50} of 1.5 mg/L to *P. subcapitata*, while this endpoint was 100 mg/L in case of N^4-acetyl-SMX. [4] Moreover, reduced toxicity of N^4-acetyl-SMX and N^4-hydroxy-acetyl-SMX (TP312) to *Vibrio fischeri* was demonstrated.[5] In our previous study, we also reported reduced toxicity of TP254 and TP256 to SMX-susceptible *Escherichia coli* K-12 strain [6]. In general, we cannot observe a clear trend in the predicted LC_{50} and EC_{50} values for SMX and the observed TPs in the selected aquatic organisms.

Supplementary tables

Table S1. The MRM transitions of the analytes of interest for targeted screening. The dwell time of each analyte was 40 ms. The ion source-dependent MS parameters which were kept constant during the whole acquisition were as follows: curtain gas (CUR): 35 psi; ion spray voltage (IS): +5,000 V; turbo spray temperature (TEM): 450°C; nebulizer gas (GS1): 60 psi; heater gas (GS2): 60 psi; CAD Gas: medium. Nitrogen was used as the curtain and collision gas.

**Analyte**	**R_t (min) [a]**	**Precursor Ion (*m/z*)**	**Product Ions (*m/z*)**	**DP (V) [b]**	**EP (V) [c]**	**CE (V) [d]**	**CXP (V) [e]**
SMX	7.02	254.0	156.0 / 108.0	55 / 55	10 / 10	22 / 32	12 / 10
N^4-AcSMX	7.38	296.1	134.1 / 198.1	90 / 90	10 / 10	26 / 36	6 / 6
SMX-d4	7.01	258.2	160.0	55	10	22	12

[a] R_t: retention time, [b] DP: declustering potential, [c] EP: entrance potential, [d] CE: collision energy, [e] CXP: cell exit potential

Table S2. *p*MRM transitions of transformation products (TPs) for targeted screening

**Analyte**	**R_t (min) [a]**	**Precursor Ion (*m/z*)**	**Product Ions (*m/z*)**	**DP (V) [b]**	**EP (V) [c]**	**CE (V) [d]**	**CXP (V) [e]**
SMX	7.02	254.0	156.0 / 108.0	55 / 55	10 / 10	22 / 32	12 / 10
N^4-AcSMX	7.38	296.1	134.1 / 198.1	90 / 90	10 / 10	26 / 36	6 / 6
SMX-d4	7.01	258.2	160.0	55	10	22	12
TP254	6.14	254.0	156.0	55	10	22	12
TP256	3.20	256.1	156.1	55	10	20	10
TP258	3.47	258.1	156.0	55	10	20	10
TP270	6.83	270.1	156.0	55	10	25	10
TP272	4.93	272.2	108.1	55	10	25	10
TP288	6.29	288.0	156.1	55	10	25	10
TP214	2.57	214.0	156.1	55	10	20	10
TP216	2.83	216.1	108.0	55	10	20	10
TP188	1.98	188.2	108.1	55	10	20	10

[a] R_t: retention time, [b] DP: declustering potential, [c] EP: entrance potential, [d] CE: collision energy, [e] CXP: cell exit potential

Table S3. QSAR analysis of SMX and its TPs using VEGA QSAR. More hazardous predicted values are denoted in red, whereas less hazardous predicted values are denoted in green.

ID	BCF model [log(L/kg)] KNN Read-across v1.1.1	*Daphnia magna* LC_{50} 48 h (mg/L) EPA v1.0.9	*Fathead minnow* LC_{50} 96 h (mg/L) EPA v1.0.10	*P. subcapitata* EC_{50} 72 h (mg/L) ProtoQSAR-Combase v1.0.1
SMX	0.26	45.8	34.1	0.135
TP254	0.15	13.2	60.2	0.112
TP256	0.20	45.4	104.8	0.132
TP258	0.20	45.4	111.8	0.132
TP270	0.17	215.5	41.2	0.140
TP272	0.65	709.7	130.0	0.128
TP288	0.75	443.9	579.5	0.129
TP214	0.11	14.6	185.5	328.8
TP216	0.10	76.6	128.8	374.1
TP188	0.52	73.6	8.7	183.4
TP312	0.86	143.5	54.4	0.104
N^4-acetyl-SMX	1.50	17.8	33.5	0.119

Table S4. Absolute peak area counts of transformation products (TPs) detected in samples over the course of successive sulfate-reducing (SRC), abiotic-oxic (AbOx) and biotic-oxic (BiOx) treatment stages.

		Peak area counts (cps)								
Sample name	Time (d)	TP254	TP256	TP258	TP270	TP272	TP288	TP214	TP216	TP188
P4-SMX1	0	9.70E+06	1.56E+07	1.10E+06	0.00E+00	0.00E+00	2.82E+05	7.33E+05	0.00E+00	7.50E+05
P4-SMX2	0	8.66E+06	2.55E+07	1.59E+06	0.00E+00	0.00E+00	3.52E+05	6.00E+05	0.00E+00	8.21E+05
P4-SMX3	0	1.06E+07	2.87E+07	1.32E+06	0.00E+00	0.00E+00	4.58E+05	7.32E+05	0.00E+00	7.10E+05
P4-SMX1	6	8.44E+06	1.46E+07	1.53E+06	0.00E+00	0.00E+00	2.62E+05	9.67E+05	1.01E+05	7.36E+05
P4-SMX2	6	1.38E+07	1.43E+07	1.97E+06	0.00E+00	0.00E+00	3.06E+05	9.39E+05	7.24E+04	7.13E+05
P4-SMX3	6	1.26E+07	1.11E+07	1.43E+06	0.00E+00	0.00E+00	3.14E+05	8.83E+05	7.73E+04	7.88E+05
P4-SMX1	12	5.55E+07	8.94E+07	1.80E+06	0.00E+00	0.00E+00	2.68E+06	9.87E+05	8.02E+04	1.55E+06
P4-SMX2	12	7.66E+07	1.07E+08	1.91E+06	0.00E+00	0.00E+00	3.57E+06	1.37E+06	9.79E+04	1.17E+06
P4-SMX3	12	9.30E+07	1.08E+08	1.94E+06	0.00E+00	0.00E+00	3.30E+06	1.13E+06	1.19E+05	1.34E+06
P4-SMX1	36	7.92E+07	1.26E+08	2.76E+06	4.10E+04	1.26E+05	2.56E+06	7.95E+05	1.66E+05	2.10E+06
P4-SMX2	36	8.71E+07	1.37E+08	2.51E+06	8.74E+04	1.14E+05	3.39E+06	7.89E+05	2.14E+05	1.95E+06
P4-SMX3	36	9.98E+07	1.41E+08	3.87E+06	6.15E+04	1.39E+05	3.72E+06	9.36E+05	1.92E+05	2.13E+06
P4-SMX1	47	8.64E+07	1.63E+08	5.25E+06	8.32E+04	1.47E+05	3.08E+06	1.38E+06	2.87E+05	2.43E+06
P4-SMX2	47	9.99E+07	1.83E+08	6.09E+06	5.19E+04	2.34E+05	3.50E+06	1.41E+06	3.16E+05	3.18E+06
P4-SMX3	47	1.08E+08	1.89E+08	6.65E+06	8.80E+04	2.33E+05	4.58E+06	1.36E+06	4.03E+05	3.59E+06

P4-SMX1	65	5.99E+07	1.26E+08	4.80E+06	1.17E+05	1.31E+05	2.02E+06	9.79E+05	3.24E+05	2.67E+06
P4-SMX2	65	7.81E+07	1.34E+08	4.91E+06	1.53E+05	1.65E+05	3.37E+06	1.25E+06	4.34E+05	2.77E+06
P4-SMX3	65	9.37E+07	1.41E+08	5.86E+06	1.02E+05	1.98E+05	3.45E+06	1.22E+06	5.18E+05	2.31E+06
P4-SMX1	93	4.52E+07	1.12E+08	5.67E+06	1.27E+05	2.16E+05	2.12E+06	1.22E+06	6.07E+05	2.09E+06
P4-SMX2	93	5.34E+07	1.11E+08	4.91E+06	1.23E+05	2.99E+05	1.85E+06	1.25E+06	8.64E+05	2.17E+06
P4-SMX3	93	6.03E+07	1.13E+08	6.34E+06	1.27E+05	3.70E+05	2.45E+06	1.39E+06	8.97E+05	1.98E+06
P4-SMX1	100	4.32E+07	5.81E+04	6.28E+06	2.01E+05	5.14E+05	1.13E+06	1.76E+06	1.74E+06	3.51E+06
P4-SMX2	100	5.06E+07	3.98E+06	5.31E+06	1.43E+05	4.70E+05	1.12E+06	1.53E+06	1.59E+06	3.42E+06
P4-SMX3	100	5.64E+07	3.97E+06	7.17E+06	2.11E+05	5.14E+05	1.46E+06	1.67E+06	2.29E+06	3.64E+06
P4-SMX1	104	4.44E+07	0.00E+00	7.13E+06	1.93E+05	4.23E+05	1.03E+06	1.53E+06	1.79E+06	3.86E+06
P4-SMX2	104	5.36E+07	0.00E+00	5.71E+06	2.49E+05	4.21E+05	1.13E+06	1.79E+06	2.04E+06	4.34E+06
P4-SMX3	104	5.57E+07	0.00E+00	7.76E+06	1.38E+05	6.98E+05	1.35E+06	1.89E+06	2.37E+06	4.69E+06
P4-SMX1	107	2.89E+07	0.00E+00	4.85E+06	1.71E+05	2.91E+05	4.21E+05	1.28E+06	1.76E+06	2.92E+06
P4-SMX2	107	3.25E+07	0.00E+00	4.06E+06	1.97E+05	3.64E+05	6.92E+05	1.10E+06	1.77E+06	3.10E+06
P4-SMX3	107	3.73E+07	0.00E+00	6.02E+06	9.95E+04	4.76E+05	8.55E+05	1.21E+06	1.74E+06	3.16E+06
P4-SMX1	112	3.63E+07	0.00E+00	5.80E+06	1.73E+05	4.58E+05	5.76E+05	1.45E+06	1.65E+06	4.14E+06
P4-SMX2	112	3.94E+07	0.00E+00	4.85E+06	1.03E+05	3.47E+05	9.94E+05	1.68E+06	1.78E+06	3.94E+06
P4-SMX3	112	4.27E+07	0.00E+00	6.85E+06	1.40E+05	3.55E+05	1.11E+06	1.53E+06	1.93E+06	3.58E+06
P4-SMX1	116	3.07E+07	0.00E+00	4.48E+06	1.14E+05	3.82E+05	4.43E+05	1.04E+06	1.99E+06	3.17E+06

P4-SMX2	116	3.09E+07	0.00E+00	3.84E+06	1.10E+05	4.87E+05	5.06E+05	1.31E+06	1.81E+06	2.95E+06
P4-SMX3	116	3.91E+07	0.00E+00	5.00E+06	1.26E+05	3.61E+05	8.67E+05	1.21E+06	1.95E+06	3.21E+06
P4-SMX1	122	3.70E+07	0.00E+00	6.90E+06	1.40E+05	5.66E+05	6.95E+05	1.71E+06	2.26E+06	4.88E+06
P4-SMX2	122	4.55E+07	0.00E+00	6.13E+06	1.66E+05	6.75E+05	5.40E+05	1.70E+06	2.39E+06	4.67E+06
P4-SMX3	122	4.76E+07	0.00E+00	8.92E+06	1.42E+05	6.43E+05	1.31E+06	2.10E+06	2.41E+06	4.23E+06
P4-SMX1	129	3.98E+07	0.00E+00	7.20E+06	1.04E+05	5.54E+05	5.91E+05	2.00E+06	2.71E+06	5.24E+06
P4-SMX2	129	4.59E+07	0.00E+00	5.74E+06	1.17E+05	6.90E+05	5.28E+05	1.85E+06	2.78E+06	5.06E+06
P4-SMX3	129	5.38E+07	0.00E+00	9.37E+06	1.90E+05	6.37E+05	1.11E+06	1.89E+06	3.00E+06	4.88E+06
P4-SMX1	139	5.51E+07	0.00E+00	8.75E+06	2.16E+05	5.59E+05	4.28E+05	1.75E+06	2.73E+06	5.37E+06
P4-SMX2	139	5.47E+07	0.00E+00	6.55E+06	2.86E+05	5.39E+05	5.99E+05	1.75E+06	2.74E+06	5.29E+06
P4-SMX3	139	7.18E+07	0.00E+00	1.10E+07	1.98E+05	6.69E+05	1.62E+06	2.41E+06	2.70E+06	5.18E+06
P4-SMX1	141	3.40E+07	0.00E+00	6.70E+06	1.44E+05	6.08E+05	0.00E+00	1.62E+06	3.01E+06	4.70E+06
P4-SMX2	141	3.62E+07	0.00E+00	5.95E+06	1.77E+05	7.49E+05	0.00E+00	1.87E+06	3.07E+06	4.69E+06
P4-SMX3	141	4.95E+07	0.00E+00	6.45E+06	1.25E+05	8.44E+05	0.00E+00	1.97E+06	3.18E+06	4.89E+06
P4-SMX1	145	2.39E+07	0.00E+00	4.66E+06	1.32E+05	5.20E+05	0.00E+00	9.31E+05	2.78E+06	4.30E+06
P4-SMX2	145	2.15E+07	0.00E+00	4.21E+06	8.80E+04	5.24E+05	0.00E+00	9.54E+05	3.17E+06	4.60E+06
P4-SMX3	145	3.23E+07	0.00E+00	5.56E+06	1.14E+05	8.79E+05	0.00E+00	1.07E+06	3.32E+06	4.16E+06
P4-SMX1	149	1.08E+06	0.00E+00	3.61E+05	0.00E+00	0.00E+00	0.00E+00	7.20E+04	2.48E+05	2.22E+05
P4-SMX2	149	8.44E+05	0.00E+00	2.16E+05	0.00E+00	0.00E+00	0.00E+00	3.18E+04	2.62E+05	1.63E+05

P4-SMX3	149	1.79E+06	0.00E+00	3.94E+05	0.00E+00	0.00E+00	0.00E+00	5.95E+04	2.59E+05	2.08E+05
P4-SMX1	152	4.39E+04	0.00E+00	0.00E+00	0.00E+00	0.00E+00	0.00E+00	0.00E+00	0.00E+00	0.00E+00
P4-SMX2	152	2.22E+04	0.00E+00	0.00E+00	0.00E+00	0.00E+00	0.00E+00	0.00E+00	0.00E+00	0.00E+00
P4-SMX3	152	0.00E+00	0.00E+00	0.00E+00	0.00E+00	0.00E+00	0.00E+00	0.00E+00	0.00E+00	0.00E+00
P4-SMX1	155	2.08E+05	0.00E+00	0.00E+00	0.00E+00	0.00E+00	0.00E+00	0.00E+00	0.00E+00	0.00E+00
P4-SMX2	155	1.06E+05	0.00E+00	0.00E+00	0.00E+00	0.00E+00	0.00E+00	0.00E+00	0.00E+00	0.00E+00
P4-SMX3	155	2.54E+05	0.00E+00	0.00E+00	0.00E+00	0.00E+00	0.00E+00	0.00E+00	0.00E+00	0.00E+00
P4-SMX1	163	0.00E+00	0.00E+00	0.00E+00	0.00E+00	0.00E+00	0.00E+00	0.00E+00	0.00E+00	0.00E+00
P4-SMX2	163	0.00E+00	0.00E+00	0.00E+00	0.00E+00	0.00E+00	0.00E+00	0.00E+00	0.00E+00	0.00E+00
P4-SMX3	163	0.00E+00	0.00E+00	0.00E+00	0.00E+00	0.00E+00	0.00E+00	0.00E+00	0.00E+00	0.00E+00
P4-SMX1	171	0.00E+00	0.00E+00	0.00E+00	0.00E+00	0.00E+00	0.00E+00	0.00E+00	0.00E+00	0.00E+00
P4-SMX2	171	0.00E+00	0.00E+00	0.00E+00	0.00E+00	0.00E+00	0.00E+00	0.00E+00	0.00E+00	0.00E+00
P4-SMX3	171	0.00E+00	0.00E+00	0.00E+00	0.00E+00	0.00E+00	0.00E+00	0.00E+00	0.00E+00	0.00E+00

3

Supplementary figures

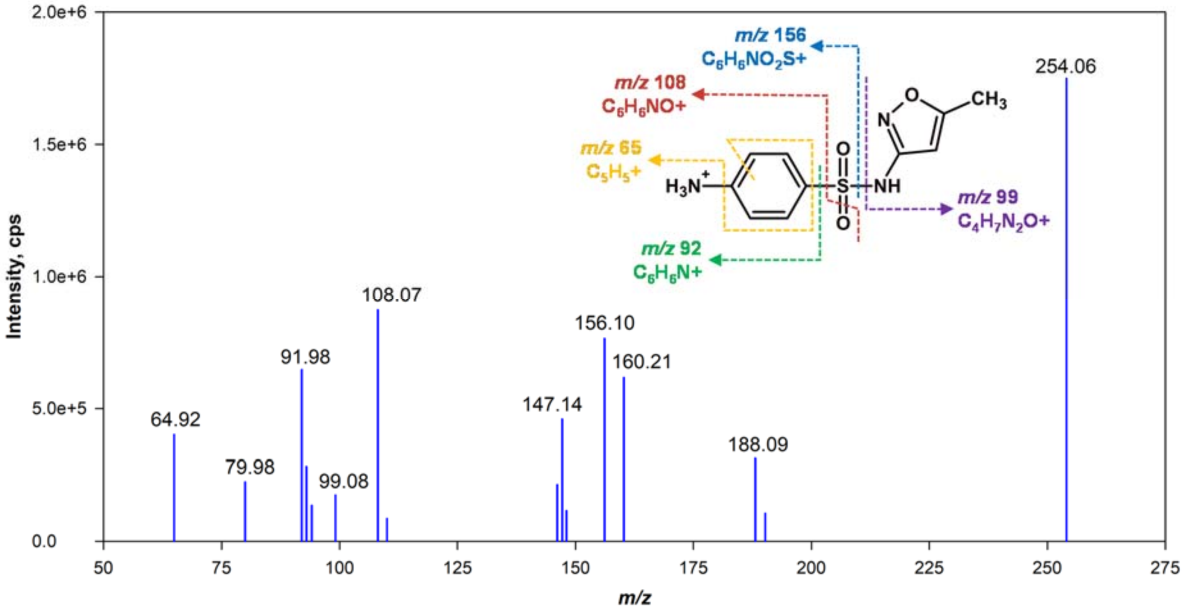

Figure S1. Mass spectrum of sulfamethoxazole (SMX) and structural analysis diagram.

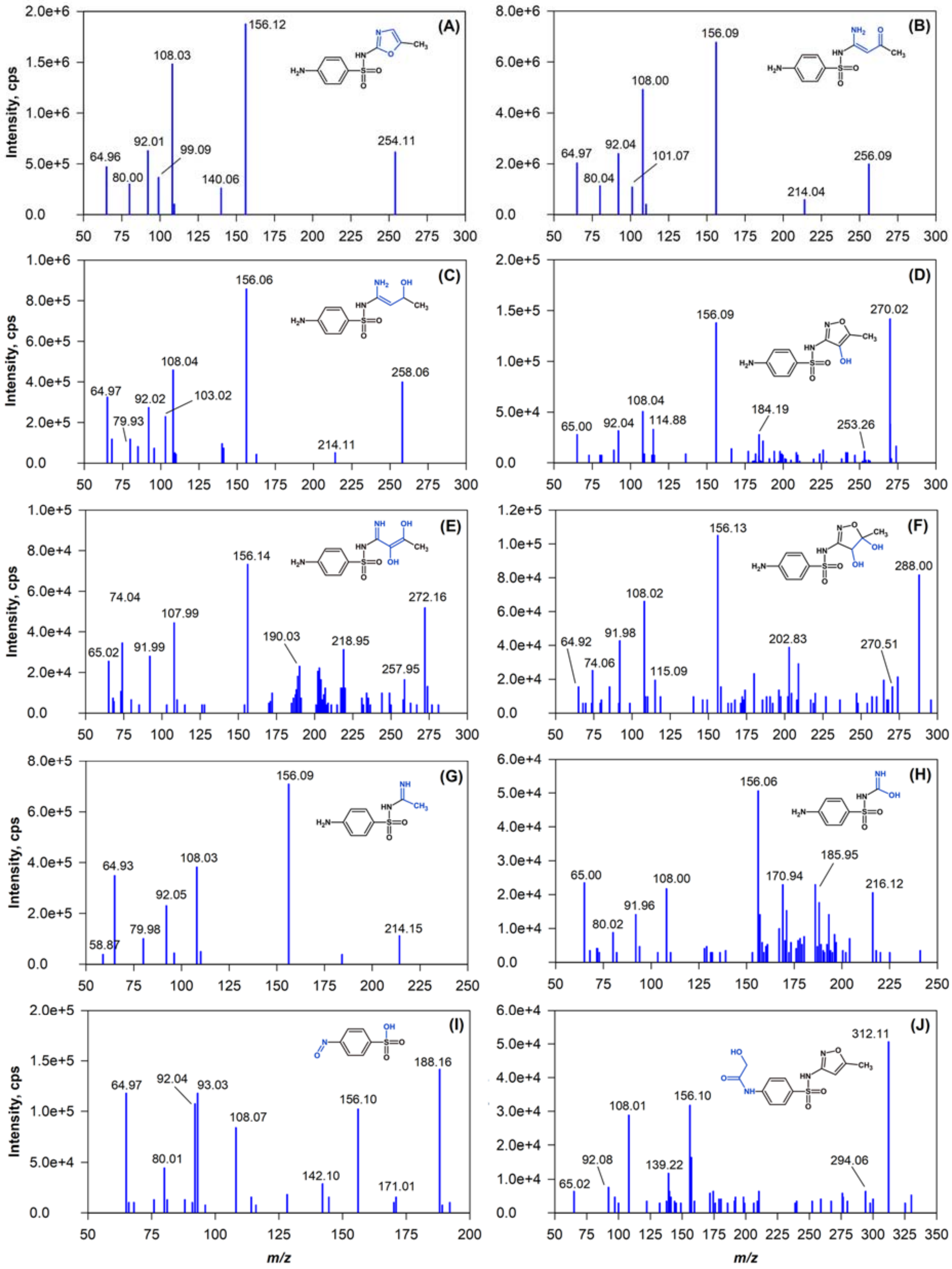

Figure S2. Mass spectra of the detected transformation products (TPs) of SMX in passage 3 (P3) enrichment cultures under nitrate- or sulfate- reducing conditions.

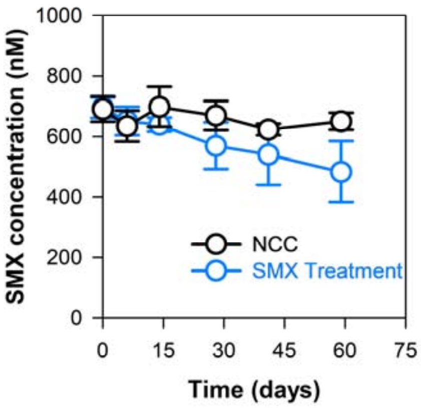

Figure S3. SMX biotransformation with the cultures from passage 4 (P4) under nitrate-reducing conditions (NRC). Values represent means ± standard deviations, *n*=3.

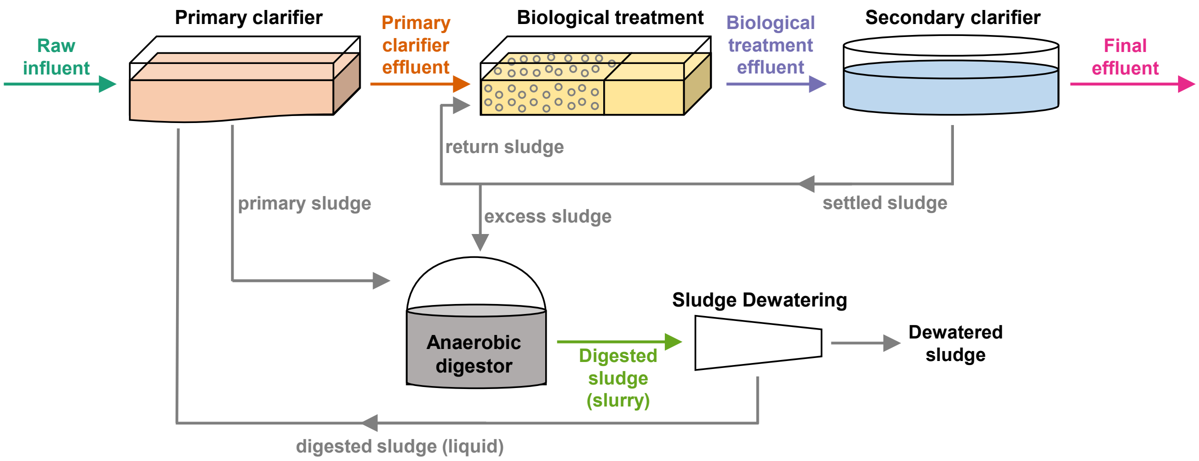

Figure S4. Simplified process diagram of Rosental WWTP in Leipzig, Germany. Raw influent is the inflow of sewage after mechanical cleaning (belt screening and sand trap). The primary clarifier unit removes suspended solids at a low flowrate from the wastewater stream, while primary sludge is transported to the anaerobic digestor. Subsequently, in the biological treatment, consisting of oxic-anoxic cascading system, wastewater undergoes nitrification-denitrification for the biological removal of nitrogen and formation of activated sludge. The secondary clarifier is used to separate the wastewater-activated sludge mixture. The settled sludge is recirculated back to the biological treatment. After this, treated wastewater leaves the secondary clarifier and discharged into the river “Neue Luppe”. Excess sludge collected from the secondary clarifiers is treated anaerobically under mesophilic conditions in the anaerobic digestor with an average sludge retention time of 30 d. The digested sludge is mechanically dewatered, and the liquid is recirculated back to the primary clarifier.

Supplementary references

(1) Edgar, R. C. Search and clustering orders of magnitude faster than BLAST. *Bioinformatics* **2010**, *26* (19), 2460-2461. DOI: 10.1093/bioinformatics/btq461

(2) Ding, C.; Adrian, L.; Peng, Y.; He, J. 16S rRNA gene-based primer pair showed high specificity and quantification accuracy in detecting freshwater *Brocadiales* anammox bacteria. *FEMS Microbiol Ecol* **2020**, *96* (3). DOI: 10.1093/femsec/fiaa013

(3) Wang, Q.; Garrity, G. M.; Tiedje, J. M.; Cole, J. R. Naive Bayesian classifier for rapid assignment of rRNA sequences into the new bacterial taxonomy. *Appl Environ Microbiol* **2007**, *73* (16), 5261-5267. DOI: 10.1128/AEM.00062-07

(4) Eguchi, K.; Nagase, H.; Ozawa, M.; Endoh, Y. S.; Goto, K.; Hirata, K.; Miyamoto, K.; Yoshimura, H. Evaluation of antimicrobial agents for veterinary use in the ecotoxicity test using microalgae. *Chemosphere* **2004**, *57* (11), 1733-1738. DOI: 10.1016/j.chemosphere.2004.07.017

(5) Majewsky, M.; Wagner, D.; Delay, M.; Brase, S.; Yargeau, V.; Horn, H. Antibacterial activity of sulfamethoxazole transformation products (TPs): general relevance for sulfonamide TPs modified at the para position. *Chem Res Toxicol* **2014**, *27* (10), 1821-1828. DOI: 10.1021/tx500267x

(6) Ouyang, W. Y.; Birkigt, J.; Richnow, H. H.; Adrian, L. Anaerobic transformation and detoxification of sulfamethoxazole by sulfate-reducing enrichments and *Desulfovibrio vulgaris*. *Environ Sci Technol* **2021**, *55* (1), 271-282. DOI: 10.1021/acs.est.0c03407
